# Chronic radiation proctitis: tricks to prevent and treat

**DOI:** 10.1007/s00384-015-2289-4

**Published:** 2015-07-23

**Authors:** Ben G. L. Vanneste, Lien Van De Voorde, Rogier J. de Ridder, Evert J. Van Limbergen, Philippe Lambin, Emile N. van Lin

**Affiliations:** Department of Radiation Oncology (MAASTRO Clinic), GROW-School for Oncology and Developmental Biology, Maastricht University Medical Center, P.O. Box 3035, 6202 NA Maastricht, The Netherlands; Department of Gastroenterology, Maastricht University Medical Center, Maastricht, The Netherlands

**Keywords:** Radiotherapy, Radiation proctitis, Prevention, Treatment

## Abstract

**Objective:**

The purpose of this study was to give an overview of the measures used to prevent chronic radiation proctitis (CRP) and to provide an algorithm for the treatment of CRP.

**Methods:**

Medical literature databases including PubMed and Medline were screened and critically analyzed for relevance in the scope of our purpose.

**Results:**

CRP is a relatively frequent late side effect (5–20%) and mainly dependent on the dose and volume of irradiated rectum. Radiation treatment (RT) techniques to prevent CRP are constantly improving thanks to image-guided RT and intensity-modulated RT. Also, newer techniques like protons and new devices such as rectum spacers and balloons have been developed to spare rectal structures. Biopsies do not contribute to diagnosing CRP and should be avoided because of the risk of severe rectal wall damage, such as necrosis and fistulas. There is no consensus on the optimal treatment of CRP. A variety of possibilities is available and includes topical and oral agents, hyperbaric oxygen therapy, and endoscopic interventions.

**Conclusions:**

CRP has a natural history of improving over time, even without treatment. This is important to take into account when considering these treatments: first be conservative (topical and oral agents) and be aware that invasive treatments can be very toxic.

## Introduction

Radiation injury to the rectum represents a feared complication of radiotherapy (RT) in urological, gynecological, and gastrointestinal malignancies (prostate, urinary bladder, cervix, uterus, and anus). Chronic radiation proctitis (CRP) is a relatively frequent late (after 3–6 months) side effect that affects 5–20 % of cancer patients [1–3]. The probability of developing the injury is related to the volume of rectum irradiated, total RT dose, RT technique, and dose per fraction [4]. Also, individual patient factors can influence the susceptibility to CRP: comorbidity of vascular disease, diabetes, connective tissue disease or inflammatory bowel disease, specific conditions such as smoking, and concomitant chemotherapy [5, 6]. Published nomograms based on patient risk factors (use of anti-coagulants, hormonal therapy, or anti-hypertensives; presence of diabetes or hemorrhoids, and a history of pre-RT abdominal surgery) have been predictive for CRP in prostate cancer [7, 8].

Until now, no optimal management has been defined for CRP. A variety of therapeutic modalities is available ranging from oral agents to endoscopic interventions. The aim of this article is to summarize the measures being developed for the prevention of CRP and to present a practical algorithm for the treatment of CRP based on a review of the literature.

## Symptoms

RT can cause both early (acute) and late (chronic) side effects [9]. Acute side effects by definition occur up to 3 months after RT and are usually self-limiting. Chronic side effects occur 3–6 months after RT or even years later. The side effects of RT are scored in five groups (see Table 1) according to the National Cancer Institute Common Terminology Criteria for Adverse Events 4.0 (CTCAE) [10]. Acute side effects include diarrhea, mucus discharge, urgency, tenesmus, and uncommonly bleeding [5]. Similar symptoms are seen in patients with chronic CRP, but bleeding is the most common symptom, with potential iron-deficiency anemia that requires blood transfusions. In addition, patients may have symptoms of obstructed defecation due to strictures with symptoms of constipation, rectal pain, urgency, and, rarely, fecal incontinence due to overflow [5]. In a series of studies, Andreyev et al. identified 23 different symptoms that develop after pelvic RT [11–13].

**Table 1:** Radiation proctitis according to the ‘common toxicity criteria’, version 4

Grade CRP	Symptoms
1	Rectal discomfort; intervention not indicated
2	Symptoms (rectal discomfort, passing blood or mucus); medical intervention indicated; limiting instrumental ADL
3	Severe symptoms; fecal urgency or stool incontinence; limiting self-care ADL
4	Life-threatening consequences; urgent intervention indicated
5	Death

## Pathogenesis

CRP results from progressive epithelial atrophy and fibrosis associated with obliterative endarteritis, chronic mucosal ischemia, submucosal fibrosis, and new vessel formation, which have been shown to lead to clinical symptoms [14]. This is not further discussed here because they go beyond the scope of this article and can be found elsewhere [14, 15].

## Diagnosis

CRP should be suspected in any patient who has had pelvic RT and presents with the symptoms mentioned above, even if the radiotherapy took place years ago. Diagnosis by endoscopy is important to exclude other causes of proctitis (infectious colitis, inflammatory bowel disease, diversion colitis, ischemic colitis, diverticular colitis) and a second malignancy [15].

**Fig. 1:**
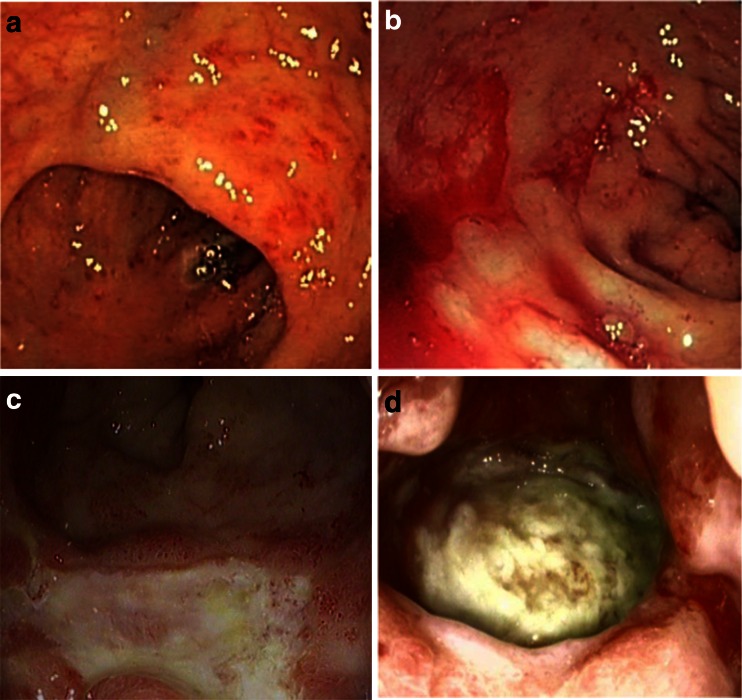
Endoscopic features illustrating the different grades of CRP at the anterior rectum wall. **a** shows CRP with edematous and multiple non-confluent telangiectatic lesions, **b** demonstrates a predominantly bleeding form of CRP, **c** illustrates necrosis with multiple confluent telangiectatic lesions, and **d** shows an ulcer

Endoscopy is also important to determine the extent and severity of CRP. There are three main forms of endoscopic findings in CRP: inflammation predominant form (I-CRP) (edema, mucosal pallor, and ulcer), bleeding predominant form (B-CRP) (friability, spontaneous hemorrhage, and telangiectasia), and a mixed form (with features from both I-CRP and B-CRP) (Fig. 1a–d) [15–17]. The endoscopic classification of CRP is usually analyzed by the Vienna Rectoscopy Score (VRS) to describe rectal mucosa [18]. The VRS divides the inner rectal mucosa into 12 mucosal areas. Furthermore, each area is scored on the presence and grading of telangiectasia (Grade 0–3), congested mucosa (Grade 0–3), ulceration (Grade 0–4), stricture (Grade 0–4), and necrosis (Grade 0–1). However, other scoring systems also exist [19].

Rectal wall biopsies should be avoided as they may initiate chronic, poorly healing wounds. A number of studies have also described fistula formation over the prostate following rectal biopsies [20–22]. Therefore, a biopsy is only justified if a malignancy is suspected or in case of important therapeutic consequences. In these cases, biopsies should be taken from the posterior and lateral rectal walls to avoid the anterior irradiated high-dose areas [20]. In conclusion, biopsies do not really contribute to the diagnosis of CRP and should be avoided.

## Prevention

Excluding the rectum from the irradiation fields to prevent CRP has a high priority in RT. Optimizing the RT planning by using planning constraints reduces the irradiated rectal volume and consequently decreases the risk of rectal toxicity [23].

There is increasing evidence supporting the role of genetic variants in the development of RT-induced toxicity [24]. Recently, the first replicated genetic associations for adverse reactions to RT were reported [25]. Active research to identify high-risk patients is based on genetic biomarkers [26] that could allow radiotherapists to select patients for which extra care should be taken to decrease the dose to the rectum.

### Different RT techniques

The use of modern RT techniques (intensity-modulated RT) and the use of implanted fiducial markers into the prostate (image-guided RT) minimize the dose of radiation to the rectum while maximizing the dose to the prostate [27–29].

Newer RT techniques which utilize heavy particles such as protons and carbon ions are currently being developed and tested to improve outcomes with reduced toxicity [30, 31]. Carbon ions seem to be better protective than protons which can be explained by the steeper dose gradients achieved by heavier particles [32]. Although these methods have the potential to deliver optimal doses of radiation to the tumor with only minimal exposure to the surrounding normal tissues, the long-term outcomes are not yet clear.

### Medication

The use of medical therapy (amifostine, sucralfate, 5-aminosalicylic acid, or sulphasalazine) to prevent the development of CRP has only a minimal effect and is not widely used [33–36]. Placebo-controlled phase III trials have shown no benefit from either topical or oral sucralfate [37]. However, higher doses of amifostine are described as tolerable and as having a better protective effect against the early and late short-term effects of RT [38].

Newer insights have revealed that synbiotics and microbiotics can be used to manipulate the intestinal flora to prevent and treat CRP [39, 40]. Further research is needed to confirm those preliminary data.

### Rectum spacer

**Fig. 2:**
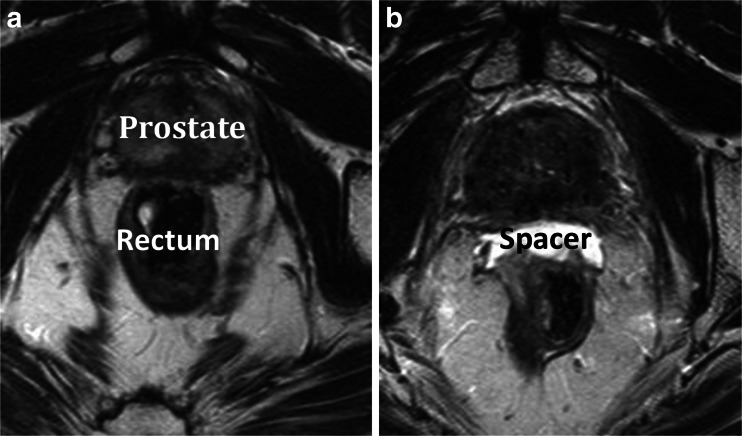
Axial T2-weighted magnetic resonance images of a patient with a hydrogel spacer before injection (**a**) and after injection (**b**)

Devices have been developed to spare rectal structures [41]. These can be divided into endorectal balloons and relatively novel rectum spacers. Endorectal balloons are inserted into the rectum for each daily treatment fraction to increase the distance from the dorsal rectal wall to the prostate. Although the anterior anorectal wall is pushed towards the prostate, the overall effect proved to be beneficial in 3D-conformal RT and intensity-modulated RT [42]. Rectum spacers are implanted as a tissue filler into the anterior perirectal fat to separate the rectum from the prostate (Fig. 2). Increasing the prostate-rectum distance displaces the rectal wall away from the prostate and out of the regions of high-dose RT. The overall effect is a reduction in the maximum dose to the rectum and the total volume of irradiated rectum. The implantation of such rectum spacers is typically performed transperineally under real-time transrectal ultrasound guidance. The insertion procedure can be performed under local (with or without sedation), spinal, or general anesthesia [43]. The implanted rectum spacer remains in place over the course of the RT treatment, and the spacer biodegrades naturally within 6 months after implantation [44]. Different types of rectum spacers have been developed: an absorbable hydrogel, a hyaluronic acid, a collagen, and a saline-filled balloon [44–46]. Several studies are available on the dosimetry, acute outcome, and cost-effectiveness of a rectum spacer; however, no long-term results are available yet [47–56].

## Treatment

A wide variety of interventions have been tried for treating CRP. There have been no large controlled randomized trials to evaluate the treatment of CRP. Also, several studies have resulted in ambiguous outcome measurements, showing no structured outcome measurement to describe and compare the findings from different trials. Some studies used the VRS scoring system to evaluate treatments; others used different clinical outcomes. Thus, experience is derived mostly from small clinical trials, expert opinions, and case reports [57, 58]. Interventions can broadly be categorized into medical therapies, endoscopic therapies, and surgical interventions. Medical therapy is the main stay of treatment for I-CRP. Endoscopy is the main treatment modality for B-CRP if the bleeding is affecting quality of life [15]. It is very important to realize, when considering invasive treatments, that CRP can improve over time without any active treatment [59].

**Fig. 3:**
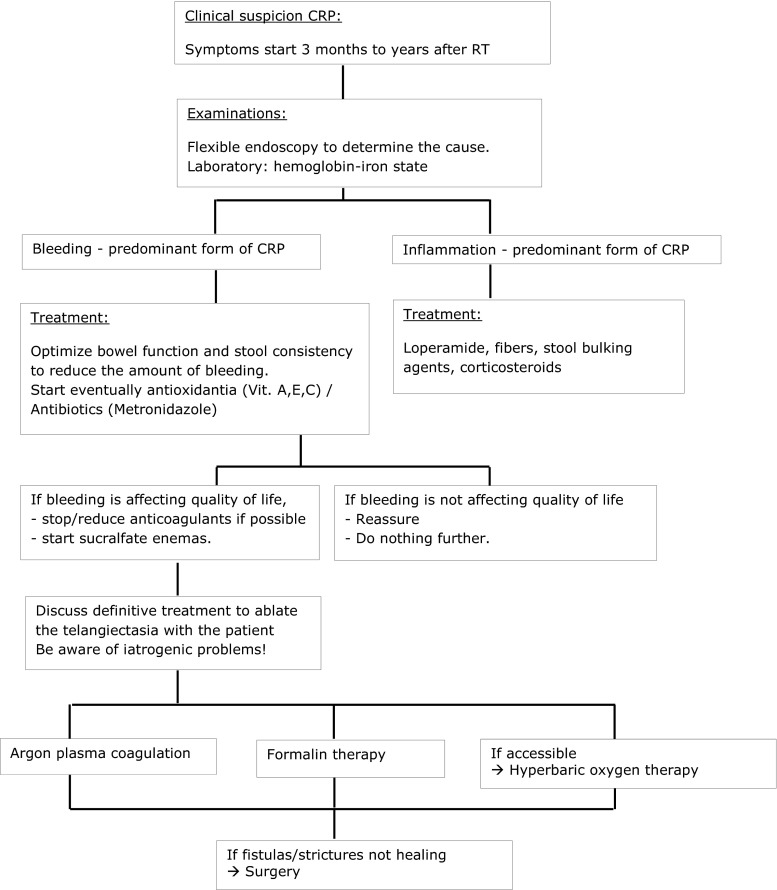
Algorithm for treatment of CRP

In patients with CRP, treatment should be based upon the pattern and severity of symptoms and experience at the treatment center. A treatment algorithm is presented in Fig. 3 for this purpose. For patients with minor symptoms that do not affect their quality of life, no treatment may be indicated because CRP has a natural history of improving over time without treatment. For patients with I-CRP, Andreyev et al. published a treatment guide that recommends loperamide, fibers, stool-bulking agents, and corticosteroids [13]. Patients with B-CRP and physical complaints of anemia (dyspnoe d’effort, palpitations, fatigue) should be monitored for anemia and where appropriate given iron supplements or blood transfusion. If necessary, endoscopic treatment is also indicated [60]. B-CRP is the most common form, and therefore, most studies have concentrated and published on B-CRP.

### Medical treatment of B-CRP

Medical treatments with level I evidence of benefits in small randomized trials are listed here: sucralfate enema [61, 62], metronidazole [63], vitamin A [64], and hyperbaric oxygen therapy (HOT) [65].

#### Sucralfate enema

Sucralfate is an aluminum salt that adheres to mucosal cells and stimulates prostaglandin production, producing cytoprotective effects. It has been used in the treatment of peptic ulcers [66].

In a prospective randomized trial, Kochhar et al. reported 37 patients with RT-induced CRP who were assigned to a 4-week course of sulfasalazine (3 g/day) plus prednisolone enemas (20 mg 2×/day) or sucralfate enemas (2 g 2×/day) [61].

Kochhar et al. subsequently reported in a prospective study on 26 patients with moderate to severe CRP who were treated with 20-ml sucralfate enemas twice daily until bleeding stopped or failure of therapy was acknowledged. Response to the therapy was considered good when the severity of bleeding improved by two grades. This was observed in 77 % of patients [62]. Kochhar et al. concluded that sucralfate enemas give a better clinical response and are better tolerated.

Although placebo-controlled randomized trials are needed to fully assess efficacy, sucralfate is recommended as the preferred mode of short-term treatment.

#### Metronidazole

Anti-microbial agents could be effective in CRP because of their immune-modulatory effects [67] and selective toxicity to microorganisms that contribute to the pathogenesis of CRP [68].

Cavcic et al. reported on 60 patients with CRP who received mesalazine (1 g 3×/day) and a betamethasone enema (1/day during 4 weeks) with or without oral metronidazole (400 mg 3×/day) [63]. The addition of metronidazole was associated with a reduction in rectal bleeding, diarrhea, and ulcers at 4 weeks, 3 months, and 12 months. This trial suggested that metronidazole can improve symptoms and mucosal healing in combination with anti-inflammatory treatments.

#### Vitamin A/E/C

Antioxidants were suggested to have cytoprotective effects by reducing cellular oxidative stress following radiation injury to intestinal tissue [69].

Ehrenpreis et al. reported on a prospective double-blind trial including 19 patients with CRP 6 months after RT who were randomized to receive oral vitamin A (10,000 IU 2×/day, during 90 days) or a placebo. Vitamin A significantly reduced rectal symptoms of CRP [64].

Kennedy et al. reported on 20 patients treated for symptomatic CRP with oral vitamin E (400 IU 3×/day) and vitamin C (500 mg 3×/day). Bleeding resolved in 4 of 11 patients, and diarrhea resolved in 50 % of patients [70].

#### Hyperbaric oxygen therapy

HOT involves patients breathing pure oxygen in a pressurized room or tube. In a HOT chamber, the air pressure is increased to three times higher than normal air pressure [71]. Under these conditions, the lungs can gather more oxygen than at normal air pressure. This higher oxygenated blood may be beneficial because it inhibits bacterial growth and stimulates the release of growth factors and stem cells, which promotes wound healing. It may even reverse progressive changes caused by RT and may improve other symptoms such as urinary problems [72, 73].

Clarke [65] performed a controlled randomized trial with groups that were randomized to HOT at 2.0 atm absolute or air at 1.1 atm absolute. HOT significantly improved the healing responses in patients with refractory CRP, generating an absolute risk reduction of 32 % (number needed to treat, 3) [65].

A Cochrane review revealed a significantly increased chance of improvement or cure following HOT for CRP (RR 1.72; 95 % CI 1.0 to 2.9, *p* 0.04) [74].

Unfortunately, hyperbaric oxygen facilities are not always available, so patients may need to travel long distances to their nearest unit, and treatments are time-consuming (60–120 min for 30–70 sessions) and expensive [75].

### Endoscopic treatment

A variety of endoscopic therapies is available for rectal bleeding caused by CRP including argon plasma coagulation (APC), topical formalin, laser, heater, and bipolar probes. There is no level I evidence of benefit in randomized trials. The goal of endoscopic treatments of CRP is to control bleeding. Endoscopic treatments may require multiple procedures and can have very significant adverse effects [60]. Due to a known high-potential risk of fistulas and ulcerations in the first 2 years after RT, we advise that all endoscopic treatments should be performed by an experienced gastroenterologist with particular awareness of post-RT rectal injury in close collaboration with a pelvic radiation oncologist [76].

#### Argon plasma coagulation

APC is a form of electrocautery, in which a monopolar diathermy is transmitted to the target tissue through an ionized gas in a non-contact fashion (0.8–3.0 mm from the target) [77]. APC is considered by many gastroenterologists as the treatment of choice for CRP [78–81]. However, it should be used with caution in this patient group. Complications such as bowel explosions following the use of APC in inadequately prepared bowels have been described but are preventable [82]. Other severe side effects, such as the occurrence of deep ulceration [83, 84], fistulation [85], stricture formation [86–88], bleeding [83, 84, 89], perforation [83], and severe and sometimes chronic pain [80, 89, 90], reflect the risk of any therapy in chronically ischemic tissues. Rectal ulcers after APC when used for CRP are observed in approximately 26 % of patients, in one series, even up to 52 % [91–93]. Together with restricting argon flow rates and wattage, a very precise and brief application of the argon catheter could potentially reduce complication rates [94]. In specialist centers, serious complications of previous APC treatment in this patient group continue to be seen regularly [60].

Swan et al. presented a complete resolution of bleeding in 72 % of 50 patients who had bleeding CRP [79]. Thirty-four percent of the patients experienced short-term, self-limiting complications; 2 % experienced a long-term complication. The setting was a tertiary referral hospital, where only dedicated and experienced gastroenterologists were involved in post-RT rectal injury.

#### Topical formalin

Formalin seals fragile neovasculature in radiation-damaged tissues to prevent further bleeding through chemical cauterization [95, 96]. This treatment is simple to perform, but a severe disadvantage is a chemical burn to the skin if there is spillage [60].

There are several small retrospective studies on the use of formalin. These studies used a variety of formalin application techniques, from irrigation to direct application, and formalin concentrations, from 3.6 to 10 % [97]. The short-term success rate of this technique ranged from 60 to 100 % [98–109]. However, the procedure is not risk free and may induce major complications such as acute colitis [110].

Yeoh et al. showed that APC and topical formalin had comparable efficacy in the durable control of rectal bleeding associated with CRP but had no beneficial effect on anorectal dysfunction [111]. However, more authors reported that APC may be more effective in treating CRP as compared with formalin therapy [58, 89, 112].

#### Laser

The argon and neodymium/yttrium aluminum garnet (Nd:YAG) laser has been used to coagulate bleeding vessels throughout the gastrointestinal tract [113]. A study that included 65 patients treated with an Nd:YAG laser found an improvement in symptoms in 78 % of patients (range, 58 to 87 %) [114]. However, the laser is expensive and not widely available.

Assessing the effectiveness of these interventions is complicated by the small number of patients included in many trials, the lack of a control arm, and the fact that the natural history of CRP is to improve over time without treatment.

### Surgery

Surgery is considered as a last resort for patients with CRP and should be reserved for those who are found to have a stricture, permanent bleeding, perforation, or a fistula that is not responsive to the medical and endoscopic approaches [15, 115]. Surgical treatment options include excision, urinary and fecal diversion (diverting stoma), and reconstruction of a coloanal J reservoir [116]. Severe postoperative complications can occur such as sepsis, wound dehiscence, bowel obstruction, and de novo rectal fistula [76]. Yegappan et al. reported a 3 % postoperative mortality rate [117].

Fischer et al. concluded that 51 % of their participants had a fair outcome, 34 % had slight or moderate symptoms, and 14 % had disabling symptoms [118]. Lane et al. revealed that good outcomes can be expected in properly selected patients [119]. Turina et al. determined that the best results were found in patients presenting with colorectal anastomotic and primary bowel strictures as their main complication, while most patients with severe CRP and very distal strictures required permanent diversion [120].

## Conclusion

CRP is a commonly observed late side effect of pelvic RT and can occur even years after treatment. First of all, care should be taken to minimize the risk of CRP by improving RT techniques (IMRT, IGRT) or to implement new devices to spare the rectum (spacers, balloons). On the basis of the available knowledge, we constructed a practical management algorithm (Fig. 3). The literature generally recommends a flexible endoscopy to determine the cause. Biopsy, and especially anterior rectal biopsy, within the first years of RT should be avoided, because this augments the risk of a fistula and is not likely to provide any relevant information.

There are three main forms of CRP: I-CRP, B-CRP, and a mixed form. I-CRP responds well to loperamide, fibers, stool-bulking agents, and corticosteroids. B-CRP is often self-limiting and responds well to conservative management; it is advisable to stop anti-coagulants, if possible, and start with antioxidants (vitamin A, E, C) and/or antibiotics (metronidazole). If no response is observed, patients should be started on sucralfate enemas. In severe cases, with persistent bleeding, chemical (formalin) or thermal (coagulation) treatments are successful. If HOT is available, it may also be a good option. Surgery should be considered as the last resort and is only indicated if fistulas and strictures are not healing. Although surgery can lead to significant improvements, it also bears an increased risk of postsurgical complications. Based on the frequency of CRP, prospective controlled and larger studies are advised to increase our knowledge about both the prevention and treatment of CRP.
